# The Telegraphic Body: Dyspepsia, Modern Life, and ‘Gastric Time’ in Nineteenth-Century Medicine and Culture

**DOI:** 10.1007/s10912-024-09884-8

**Published:** 2024-09-16

**Authors:** Emilie Taylor-Pirie

**Affiliations:** University of Birmingham, Edgbaston, Birmingham, B15 2TT UK

**Keywords:** Gut health, Telegraphy, Digestion, Modern life, Gut-brain axis, Dyspepsia, Bodily rhythms

## Abstract

From Italian physician Hieronymus Mercurialis’s contention that the stomach was ‘the king of the belly’, to its promotion by the end of the nineteenth century to the ‘monarch of humanity’ in patent medicine, to Byron Robinson’s discovery of the enteric nervous system in 1907 (a mesh of neural connectivity that led him to dub the gut ‘the second brain’), there has historically been a longstanding awareness of the expansive reach of the gut in the functions of the body. In the nineteenth century, the authority of the gut and its allyship with the brain became a focus for writers thinking about the intersections of illness and ‘modern life’. In medical texts, domestic health manuals, patent medicine, and fiction, the electric telegraph in particular became a way of envisaging what we would now call the ‘gut-brain axis’. The telegraphic metaphor enabled a view of digestion as not simply a mechanical or chemical process, but one that was understood in terms of time, space, and communication. However, such a framework also suggested problems of connection that were common to both systems, emphasising not only the healthy body’s quasi-telegraphic networks but also its vulnerability to delay, disruption, and pathological incoherence. This article will explore the use of telegraphic technologies as proxies for theorising gastric connection and more broadly the concept of ‘gastric time’ as a key conceit for understanding digestion as a process that was and is subject to the idiosyncrasies of bodily rhythms.

## Introduction

In his best-selling book *Life Time* (2022), Russell Foster, professor of neuroscience and circadian rhythms at Oxford, reveals that the management of our circadian rhythms—the so-called ‘body clock’—operates at a molecular level via ‘clock genes’. A negative feedback loop which drives a cycle of clock-protein production and breakdown produces the 24-hour rhythms that structure our daily experience, coordinated by a ‘master clock’ located deep within the brain (in the suprachiasmatic nuclei or SCN). From sleep to digestion to immunity, these daily rhythms influence our worldly experiences in profound ways and, for Foster, are being compromised by ‘the modern 24/7 society’ with our seemingly tireless cultures of work (5).

The science here is new but the metaphor is not. The rhythmicity of the body, and, in particular, the idea of the body as a watch or clock, is historically prevalent in medical and social commentary. These mechanistic schematics precipitate conversations about how our bodies exist, often uneasily, within a technologically modern society, quietly underlining the disparity between biology and machine. Such analogies were given renewed significance in the nineteenth century with the institution of ‘standard time’ and the development of communications technologies like the telegraph, which collapsed time and space with new, almost instantaneous correspondence.

The image of the electric telegraph with its wires offering connection over distance joined the clock as a somatic metaphor that illustrated organic communication pathways, specifically the connection between the stomach and the brain. Enmeshed within a broader, developing understanding of nervous physiology, and continuous with new experimental opportunities in the field of digestive health, what we would now call the gut-brain axis became a central conceit in discussions of dyspepsia (a voguish complaint that rose in visibility throughout the century). Then, as now, such disruption to ‘normal’ bodily functions was seen as a disease of ‘modern life’.

Existing scholarship has focused on the conceptual intertwinement of telegraphic engineering and the emergent field of neurology—an intertwinement that influenced, many have argued, both the way technologies were developed and how people perceived their own minds and bodies. The close alliance between the telegraph and the nervous system hinged on the idea that they ‘appeared to be doing the same thing and for the same reasons’ and was understood in the context of their shared purpose: the transmission of information (Otis 2001, 13). Elizabeth Green Musselman claims that by the time the electric telegraph became popular in the 1840s and 1850s, following on from its optical predecessor, ‘the ground … had already been laid for thinking about the analogous challenges of nervous and telegraphic communication’ (2006, 192). In 1879, a writer for *The Edinburgh Review* remarked that ‘the process which is adopted when an electrician transmits messages along the telegraph wire, and that which nature pursues when signals are passed through the instrumentality of nerve impulse in the living animal body are so remarkably alike’ that the one might be used as a model for the other (ART. III–1. Manual of human and comparative histology, 58). However, such comparisons prompted a fuzzy definition of ‘information’ that was clearly problematic. Thus, the use of telegraphic technologies to think about the nervous system (and vice versa) often reached beyond both metaphor and metonym to a messier kind of linguistic and conceptual formulation.

The nineteenth century is no stranger to this kind of problem. Scholars of the field of literature and science have, since Gillian Beer’s ground-breaking work on ‘Darwin’s Plots’, examined the fraught—because shared—vocabularies and imaginations of nineteenth-century science and culture. Often concerned with relationships between form and content, scholars have examined the diverse ways that figurative language and literary forms have helped to create meaning for scientific fields of enquiry. Tita Chico (2018), for instance, sketches out the intertwinement of literary and scientific epistemologies (or ways of knowing) embodied in what she calls the ‘experimental imagination’. She argues that from at least the late-seventeenth century scientific practice relied on ‘extensive use of figurative language’, ‘literariness’ and metaphor for the textual representation and public communication of its discoveries. More than merely a communicative tool, such imaginative work actually ‘enabled new forms of understanding’ (2).

These new forms of understanding are visible in the work of scholars like Laura Otis (2001) who has examined how the concept of ‘membranes’ began to dominate late-nineteenth century science and literature, inflecting imperialist constructions of both body and nation. Devin Griffiths (2016) has argued that the emergence of the historical novel provided new comparative modes of narrative analysis, rooted in analogy, that could compellingly address the long-view of natural history. And more recently, Lorenzo Servitje (2021) has examined the ‘martial metaphor’ as a prime and naturalised lens through which medicine has historically been understood and public health practiced—in many cases indebted to, and mediated by, nineteenth-century literature. Thus, what I call the ‘telegraphic body’ joins a slew of analogical thinking in this period.

In this article, I examine how the gut fits into the model of the nervous body, extending current scholarly discussion to consider how modern labour and communication expectations mediate our approach to gut health and, in turn, ideas about what it means to be healthy. Just as the electric telegraph collapsed time and space with its promise of almost instantaneous global correspondence, the nervous-gastric connections that underpinned digestive health framed the body in spatial and temporal terms, highlighting the impact of sensory impulses operating at a distance. However, this process did not always operate smoothly, paralleling the telegraph’s frequent failure to cleanly and efficiently foster connection. In one sense, the electric telegraph appeared to offer an efficient conceptual model; however, it also signalled a different kind of figurative entanglement, illuminating the ways in which the body failed to keep pace with the demands of modern life.

Whilst the use of the telegraphic metaphor was not universal, it was nonetheless a widespread conceit that emerged at the intersections of discussions about ill health and modern life, inflecting public understandings of digestive distress. Diverse intellectual venues employed the telegraphic metaphor: from mainstream medicine to domestic health manuals, cookery books to journalism, and advice columns to satirical fiction. Just as today, commentators were concerned that modern lifestyles, which included city living, long working hours, stress, burnout, and harried eating patterns, were compromising the health of the populace. The language of the telegraphic body demonstrates not unity of opinion, but ubiquity of idea—illustrating the kind of ‘work’ that metaphors do in helping us to understand our bodies and ourselves. How might tracing the scope and limits of this metaphor shed light on the ways that we have historically understood our bodies in relation to technological modernity? What might we gain from examining the contexts in which we conflate the infrastructures of modern life with the infrastructures of the body?

## Dyspepsia: A modern disease

‘Dyspepsia’ appeared in *Phillip’s New World of Words* as early as 1706, meaning ‘a difficulty of digestion; or fermentation in the stomach and guts’ (OED, 2024). Such a definition does not, however, do justice to the cultural significance of a condition that would come to signify—in the following century—a very specific understanding of our relationship to ‘modern life’. In 1733, Scottish physician George Cheyne had published a book called *The English Malady*, in which he identified a ‘class and set of disorders’ that he associated with changing social conditions in England: with the ‘richness and heaviness of our food’, ‘inactivity and sedentary occupations’, and the ‘humour of living in great, populous, and consequently unhealthy towns’ (ii). In other words: the development of industrial modernity.

Writing in the eighteenth century, he identified these disorders as having their seat in a compromised nervous system, which he attributed to three causes: acute physical damage to the nerves (from trauma for example); chronic damage from environmental factors, such as exposure to the ‘injuries of the weather’ or ‘a bad, corrupt, too poor, or low diet’; and what we might call behavioural causes: intemperance, overindulgence, and inadequate exercise. Although Cheyne identified multiple factors capable of producing nervous disorder, he placed emphasis on the impact of diet, an element that extends across two of his three categories. Hence, he asserted that ‘what is swallow’d down, and received into the habit, is the first and chief efficient cause of all that mankind suffer in their bodies’ (25). Such a view gave further form to the age-old wisdom ‘you are what you eat’, or, as French gastronome Jean Anthelme Brillat-Savarin would express it in his much-quoted *Physiology du Gout*, ‘Tell me what thou eatest, and I will tell thee what thou art’ (1825, 14).

Whilst many scholars have associated the emergence of a more modern idea of the ‘nervous’ body in the nineteenth century with a conceptual move away from the gut and towards the head in understandings of ill-health, this change in emphasis, as James Kennaway and Jonathan Andrews (2019) have argued, did not necessarily devalue digestion. Rather, a renewed interest in the physiology of digestion and new experimental opportunities gave primacy to the gastrointestinal region in developing conceptions of nervous disorder. Evidence from lay testimony suggests that many patients ‘conceived of their stomachs as at the hub of a wide range of physical and mental symptoms’, (64) with the sensibility of nervous complaint helping to endow the close association between digestive and mental disorder with the ‘elite glamour’ of the learned in this period. Both medical and lay perspectives made room for a bidirectional relationship, such that digestive complaint could disturb the mind, whilst mental shock could derange the digestion. In 1778 Lady Louisa Stuart, for example, attributed her father’s stomach troubles to his reading of unsavoury things about himself in the newspapers, a dynamic that was rooted in the concept of ‘organic sympathy’ (Kennaway and Andrews 2019, 74).

### Organic sympathy

Organic sympathy—referring to ‘a particular and very remarkable consent between various parts of the body’ (Whytt 1765, vi)—became a medical preoccupation at the beginning of the nineteenth century, growing out of the development of eighteenth-century neurology. The principle of irritation: that one part of the body might be adversely affected by pain or damage in another, or that the whole system might be disordered by local ‘morbid action’, underpinned these discussions. And whilst this principle applied to all of the body’s organs and systems, the stomach emerged as an organ that was particularly associated with it. Sir Astley Cooper, surgeon to the king, wrote in his *Lectures on the Principles and Practices of Surgery* (first published in 1824), for example, ‘there is no organ so much affected by irritation, or sympathetic influence as the stomach’ (1835, 8).

The idea of a specifically *gastric* sympathy was championed by figures like English surgeon John Abernethy (1764–1831) (senior surgeon at St Bartholomew’s Hospital and inventor of the Abernethy digestive biscuit), who, as Ian Miller (2011) notes, drew on previous discourses of nervous and organic sympathy proposed by William Cullen (1710–1790) and Robert Whytt (1714–1766). British physician Alexander Philip (1770–1847), who published a treatise on indigestion in 1827, and French materialist François Broussais also advocated a specifically gastric sympathy, arguing that the tendency of inflammation in the gastrointestinal tract to pass ‘sympathetically’ to other organs explained the prevalence of digestive symptoms in systemic disease.

This idea underpinned a broadening definition of dyspepsia, which became associated with a diverse range of symptoms including, but not limited to, abdominal distension and tenderness, pain in the epigastrium, headache, nausea and vomiting, flatulence, diarrhoea, bad breath, dizziness, fatigue, constipation, heart palpitations, asthma and shortness of breath, nettle-rash, acne, hallucination and nightmare, lowness of spirits, anger, and nervous irritability. As a nebulous category of complaint, it even modified existing diagnoses producing, for example, ‘dyspeptic epilepsy’, ‘dyspeptic hypochondriasis’, ‘alcoholic dyspepsia’, and the ‘dyspepsia of tuberculosis’.^1^

Thus in 1859 the editors of *The Critic* were able to ‘tearfully’ acknowledge dyspepsia as the most commonly met with illness in England (A physician’s note-book, 342). Later in the century, domestic health manual *The Family Physician* declared: ‘indigestion is the prevailing and fashionable malady of civilised life,’ (1883, 354) expressing a continuation of eighteenth-century concerns about ‘fashionable diseases.’ And at the century’s end, Robert Bell writing for literary periodical, *The Butterfly*, asserted: ‘dyspepsia is as modern a malady as cancer’, characterising the dyspeptic as ‘a victim of civilisation, a martyr to the times’, (1899, 135) and decisively connecting the condition to the impositions of modern life. Throughout the nineteenth century, this association with industrial modernity found expression in new and different linguistic and conceptual formations, bolstered by the development of new communications technologies.

### Wires and nerves

The concept of the electric or electrostatic telegraph—that information might be transmitted at a distance with the help of electricity—emerged in the late 1700s. It was publicised by an anonymous article outlining the idea published in the *Scots’ Magazine* in 1753 (Wenzlhuemer 2012). In 1820, Danish physicist Hans Christian Ørsted demonstrated that a current of electricity passing through a wire exerts a turning effect on a magnetic needle placed near it, leading to much experimentation with the potential of electrical telegraphy in the following decade (most notably by Carl Friedrich Gauss and Wilhelm Weber in Gottingen in 1833, Pavel Schilling in St Petersburg at about the same time, and Carl Steinheil in Munich in 1836) (Liffen 2013). By 1846, Charles Wheatstone and William Cooke had patented and brought to market the first commercial electrical telegraphy system.

At the same time, electricity was being associated with the nervous system, both functionally, through studies in galvanism, and conceptually through metaphor. It seemed to reveal a close alliance between the communication systems of Victorian society and of Victorian bodies that informed a two-way metaphor between cables on the one hand and nerves on the other. Laura Otis has outlined how explorer Alexander Humboldt’s experience studying animal electricity encouraged him to ‘see’ telegraph cables early on in the century as ‘nerves transmitting the impulses of a society’, and she quotes James Carey in claiming that the telegraph became ‘a thing to think with’ that changed the way we understood our bodies and the world in this period (2001, 1–2). Thus, she argues, the hydraulic understanding of the nerves in the seventeenth and eighteenth centuries—recognisable in what Cheyne had called ‘liquors and fluids’—gave way to understandings mediated by the telegraph net in the nineteenth.

Cheyne had employed both the metaphor of a musician perceiving sound and harmony from the striking of keys and that of a bell in a steeple attached to hammers and ropes to conceptualise the relationship between the brain and the body. It was an analogical move that focused on the perception of sensation with some, albeit small, role for interpretation on the part of the musician, but none on the ringing of the bell. In contrast, the metaphor of the telegraph encourages us to move from ‘music’ to ‘message’ or from mere ‘impulse’ to ‘information’—allowing us to conceptualise the interplay between the brain and the body (or in this case stomach) in spatial and temporal terms that map onto the infrastructures of a modern, connected society.

## Regulating the human machine

In 1825, a writer for general interest periodical *The New Monthly Magazine* opined what they saw as the failure of the digestive apparatus of modern populations:

Our omnivorous ancestors, fearless of bile and defying indigestion made everything disappear before them …. but we pigmy-boweled performers of the present day are a squeamish and qualmy race, living in perpetual terror of the tyrant Bile, and in subjection to the night-mare Indigestion; poring over Peptic precepts, Cook’s oracles, Accum’s poison in the pot, and Philip’s *Treatise on the Stomach*, and yet after all unable to bring that eternal force of revolt and disorder, that Ireland of our bodily system, into the peaceful performance of our peristaltic duties. (Insubordination of Modern Stomachs, 37–38)

They employ the historically stormy political relationship between Great Britain and Ireland to express the opinion that modern nineteenth-century citizens had lost control of their bodies, unable to bring their stomachs under subjugation. The anonymous writer invokes the context of food adulteration—Frederick Accum published *A Treatise on Adulterations of Food and Culinary Poisons* in 1820, which sold one thousand copies within its first month of publication, exposing the adulteration of food to the general reader. Moreover, they gesture towards the multi-vocal landscape of dietetic advice by referencing Cook’s oracles. William Kitchiner published *The Cook’s Oracle* in 1817, a cookbook that dispensed health advice alongside its recipes. Throughout the century, many other cookbooks followed suit, offering not only advice on the best methods for preparing pies and cakes, but also edicts on health and domestic management, blurring the line between recipe and remedy. Four years later in 1821, Kitchiner published *Peptic Precepts*, a book full of hints to help those suffering from indigestion. Like many of his contemporaries, he highlighted the stomach as a ‘centre of sympathy’ and, like others still, compared the human frame to ‘a watch, of which the heart is the mainspring, the stomach the regulator’ (6).

Whilst the proposition that each new generation suffers more pronounced levels of gastric distress seems an ever-green one, anxieties like those aired above were newly underwritten by a mechanised concept of management that inflected nineteenth-century understandings of digestive health. In 1830, Abernethy had written that the stomach was ‘the most subject to disorders, most accessible to remedies, regulating and regulated by the motions and sensations of the whole system, and accommodating itself so as to keep all the parts in balance’ (7). His contention that the stomach was both ‘regulating and regulated’ contextualised digestion in relation to its ability to maintain order within the ‘human machine’, a metaphor that did much cultural work, especially—as Anson Rabinbach has argued—in relation to understandings of work and energy: ‘the human body and the industrial machine were both motors that transformed energy into mechanical work’ (1992, 2). Within this model, the stomach was perceived as an essential regulating mechanism, and digestion was positioned as a form of labour.

Whilst machines had, of course, abounded in the eighteenth century, there was a ‘remarkable discursive prominence’ of industrial systems in early and mid-Victorian culture, which Tamara Ketabgian argues formed a ‘cultural imaginary [that] both mimicked and creatively remade industrialism, in factory systems of the mind, the heart, the home, and the senses’ (2011, 4). I would add to this a gastric ‘factory system’ embodied in models of the body like those espoused by Manchester Royal Infirmary physician Henry Browne, who in 1865 characterised the human (in relation to digestion) as a ‘machine made on the most approved principles of mechanics, hydraulics, and pneumatics’ (679).

The ubiquitous comparison between the organic and the mechanical led many commentators to think about health as a state of ‘working order’ that was universal between bodies and might be achieved by observing particular ‘rules’. It was this idea that British physician Edward Johnson (1801–1867) invoked when he used the metaphor of the watch to outline the stomach as a ‘piece of perfect machinery’ made by a ‘Divine and unerring mechanician’ (1849, 100–101). Johnson, who ran water cure establishments in Hertfordshire, Warwickshire, and later Malvern, published *Results of Hydropathy* in 1849, in which he detailed cases of dyspepsia he’d treated at Stanstead Bury House. He asserted:

I cannot conceive how the stomach which is properly fed, and which has suffered no external injury, can (of itself) become diseased, any more than a good watch can fail to indicate the time so long as it is properly wound up, and carefully defended from external injury. (101)

Such linguistic framing set expectations about how health might be managed within a framework that sought to standardise bodily processes. This drive for standardisation (reflecting an increasing desire for statistical knowledge about the body) dovetailed with a growing appetite for dietetic advice that graced the pages of periodical magazines, cookery books, self-help tracts, and domestic health manuals. Whilst these venues often sought to offer guidance through aphorism, they also showcase an emergent awareness of allergy and intolerance, frequently citing anecdotes of adverse reactions to food like milk, eggs, and strawberries with the disclaimer: ‘one man’s meat is another man’s poison’. The refrain sat visibly but uneasily alongside tables and charts reporting with authority on the relative digestibility of individual articles of food—a trend that gained traction after a freak accident rendered digestion an observable event near the beginning of the century.

### Gastric time

In 1822, Canadian voyager, eighteen-year-old Alexis St Martin was shot at close range with a musket. The bullet ripped through his abdominal muscles, punctured a lung, broke several ribs, and tore a hole in his stomach. An American army surgeon called William Beaumont came to tend to his wound, but St Martin took a turn for the worse, developing a fever, a ‘distressing cough’, and troubled breathing. Nevertheless, against the odds—after nearly two full years of treatment—St Martin made an almost full recovery. *Almost* because there was one thing that did not fully heal and that was the hole in his stomach. What formed instead was a gastric fistula, which, when depressed with a finger, provided an aperture through which St Martin’s stomach could be seen digesting in *real time*.

Beaumont recognised the unique opportunity in front of him: a real-life window into the gut! On and off over the next nine years he carried out upwards of 175 experiments on St Martin. Many of these involved dipping articles of food into the stomach via the aperture, attached to a silk string. Though rudimentary, the experiments enabled Beaumont to measure the length of time it took for different types of food to be broken down by St Martin’s stomach acid, identifying the importance of the ‘gastric juice’, producing a language of digestibility, and popularising a new temporal concept that I will call here ‘gastric time’.

Beaumont helped to experimentally establish that digestion was both a mechanical and chemical process. His observations contributed to ongoing debates about the essential components of food—this was a century that saw the rise of gastric chemistry and the science of nutrition. Fats, carbohydrates, and proteins would soon become part of the national vocabulary, along with—by the mid-twentieth century—‘vital amines’ or vitamins. Nevertheless, what caught the popular imagination were the idiosyncratic rhythms and tempos of digestion, ensuring that all those watch metaphors would resonate with new meaning.

When the observations were published in a British edition in 1838, they prompted other physicians to follow Beaumont’s example and conduct experiments in which they too charted the digestibility (in hours and minutes) of individual articles of food. In 1865, *The London Reader* reproduced several tables from ‘authentic sources’ recording the ‘digestibility’, ‘nutritiousness’, and ‘value’ of ‘the most common articles of food’ for ‘general practical interest’. Boiled rice, the authors noted, was the easiest of digestion ‘because the quickest’, whilst beef suet was hardest of digestion, taking five and a half hours. Digestion is here understood as an almost direct product of time.

These discussions, aimed at a general reader, were often based on the work of physicians like Sir James Eyre, pupil of John Abernethy, and author of *The Stomach and its Difficulties* (1852). For Eyre, beef was the most digestible, followed by venison, game, and mutton. However, he also argued that puddings and vegetables were ‘unwholesome’ and pastry ‘poisonous’ to those with ‘weak digestive powers’, suggesting that digestibility was not only a product of the inherent properties of food but also of environment and individual constitution.

Indeed, medical writers identified a long list of circumstances that might affect digestion over and above the choice of aliment, including over or under-eating; eating too fast or too often; improper attention to the arrangement and timing of meals; mixing different kinds of food within one meal; not leaving enough time between meals; eating certain foods at the wrong time of day; not getting enough exercise; being subject to overwork or mental anxiety; consuming excessive amounts of tea or coffee; the use of condiments, alcohol, tobacco, and snuff; not getting enough sleep; and over-diluting the gastric juice with fluids.

These diverse factors (the list is not exhaustive) were framed in terms of facilitating a *timely* digestion, which was used as a proxy for health. As physician and professor of medicine, Robert Saundby (1849–1918) would reiterate in his Ingleby lecture at the century’s end, time was an important factor in digestion—the length of time that it took to digest food after a meal was thought to reveal the motor functions of the stomach, any delay in this process foreshadowed the onset of dyspepsia. Similarly, Irish physician Arthur Leared claimed (in a paper read before the Medical Society of London in 1879 and printed in the *British Medical Journal*) that digestion should ordinarily be an ‘unfelt process’, but that the stomach ‘can perform its task without sensation only within certain limits of time. If these limits be exceeded, dyspepsia will be produced’ (1879, 622).

#### The speed of modern life

This sense of gastric time was frequently contrasted with the ‘speed of modern life’, revealing tensions between the recognition of innate biological rhythms and the constructed rhythms of modern nineteenth-century industrial life. This is reflected in Leared’s appraisal of dyspepsia, the prevalence of which he attributed to the ‘rapid but characteristic manner in which [modern] meals are dispatched’ (1860, 33). He identified the ‘typical’ case study as a middle-aged man, ‘busily engaged in commercial pursuits’, implying a sensitivity to the value of time by remarking that the patient has a restless look in his eye as if ‘he thought it losing time to speak of his health’ (97–98). Here the tension between biological time and clock time is writ large. As Leared notes, this man (the sufferer of dyspepsia) ‘manages himself in some respects as if he were an iron locomotive rather than a creature of flesh and blood; indeed, his principal movements during the year may be found on certain railroad time tables’ (98). The inflexible regimented time of the railway is here in stark contrast with the idiosyncrasies of organic life.

Leared’s choice of language to describe his case study is also fraught with the material realities of an industry deeply entangled with modern concepts of time management. In the 1850s, the railway network became a key node in the establishment of ‘standard time’ when the Astronomer Royal, George Biddell Airy, proposed to distribute the Greenwich time signal through the telegraph system.

Since its inception, the electric telegraph had been closely associated with the railway; early proponents of the telegraph such as Charles Wheatstone and William Fothergill Cooke had recognised the commercial potential of such a partnership and championed telegraphy as essential to a ‘well-regulated and economic railway system’ (Morus 2000, 460). At a time when disparate communities throughout the nation typically operated on local time determined by the position of the midday sun, railway companies often adopted ‘London time’ to run their timetables. Airy’s proposal was to use the telegraph-railway network to replace multiple local temporalities with one standardised national time zone—Greenwich Mean Time (GMT).

From 1880, GMT became the legally enforceable measurement of time throughout Britain and Ireland; as Iwan Rhys Morus puts it ‘Greenwich would become the centre of a network of clocks, all working together through the electric telegraph system to sustain a standardized, centralized reckoning of time’ (466). This image provides a counterpart to the imagery from the beginning of this article of the SCN—the brain’s so-called ‘master clock’—regulating the myriad ‘tiny clocks’ operating at a molecular level throughout our bodies. The persistence of metaphors of technological connection and regulation reflects the fact that metaphors, as Alex Gomez Marin writes, are not simply historically and culturally contingent but are also ‘natural phenomena … grounded in the very bodily nature of our daily cognitive pursuits’ (2022). Although the physiological rationale was different, the semantics are broadly the same.

Nevertheless, the ideal of a railway running to standardised time, distributed instantaneously through the telegraph network, belied the labour that went into its realisation. A combination of competing financial interests, technical problems, and human error threatened to disrupt what was, in reality, a delicately balanced system that required careful management. It was perhaps this tension between the ideal and the reality that gave potency to the telegraph as an analogy for the healthy body. The language of productivity and management ubiquitous in both medicine and commerce certainly provided a semantic common ground from which commentators could reflect on the uneasy relationship between gastric and commercial time.

Leared highlights the unsuitability of the railway timetable as a model for organic labour. His case study gets no more rest than the track; for most of his life he has been ‘busily engaged in commercial pursuits’, in the ‘routine of business’. His slow digestion is a direct counterpart to his fast living. As Leared reminds us, ‘the healthy action of the stomach is dependent on the natural action of the body and mind’; those who are ‘chained to the excitement of London life’ with minds that are ‘seldom perfectly relaxed’ fall ‘ready victims’ to habitual dyspepsia (95).

#### Somatic time management

Leared was fairly representative in framing the connection between mental and digestive complaint in relation to a mismatch between the rhythms of modern life and the rhythms of the body. The domestic health manual, *The Family Physician* (1883), produced by the physicians and surgeons of the principal London hospitals, identified dyspepsia as a common fate for ‘men of business and intellectual workers who perform their tasks with hurry and worry and give neither brain nor stomach fair play’ (366). In their discussions of a variety of gut health complaints from indigestion to liver congestion to constipation, time management features as both a cause and a solution. They attribute flatulent dyspepsia, for example, to instances in which ‘a meal happens to be delayed beyond the accustomed hour’ (293) and elsewhere similarly attribute bowel complaints to the time of ingestion, claiming ‘there are many who can eat pastry in the middle of the day, but who daren’t touch it for supper or at a late dinner’ (356). Meanwhile, they frequently advise ‘due regulation of the periods for taking food’ and advocate a ‘regulated diet’ with appropriate timings between meals.

In regard to regulating the bowels, they insist that it is important to form ‘the habit of regularly paying a visit to the closet at the same time everyday’ (191). This should be done early so as to avoid ‘hurry and bustle’ and allow one to ‘devote more time and consideration to the subject’. Offering an example of a healthy morning routine, they advise the following:

Get up directly you wake, turn into your bath, and have a good sponge, then dress—no sitting about in your dressing gown—have your breakfast, take your paper, and your pipe if you like, and retire [to the w.c] for a good ten minutes or a quarter of an hour. It may be that you feel assured that your visit will be unproductive, nevertheless go. You may be unsuccessful today, and perhaps to-morrow, but in time you will succeed. At all events, you will have the satisfaction of knowing that you have done your duty. After a few weeks you will in all probability find that your bowels act with the regularity of clockwork. (191-92)

The suggestion here is that perseverance will enable the individual to bring their biological rhythms into alignment with clock-time, an enterprise that is clothed in the language of business (productivity and success), and of duty. And yet, this discourse of somatic time management coexisted with a prominent recognition of idiosyncrasy and individual difference. Even as they assert that ‘every robust healthy person should have a motion once in twenty-four hours’, they note that for some (healthy people) this happens two or three times a day or once every two to three days (191).

This tension between the desire for universal rules and the recognition of individual idiosyncrasies was widespread. Whilst the nutritive properties of food might be objectively gleaned from chemical analysis, the ‘digestibility’ of those articles was subject to bodily difference and, as amateur physiologist George Henry Lewes pointed out, ‘unless the substance can be digested, it cannot be assimilated, cannot nourish’ (1859, 115). Thus, when giving out advice on diet, many commentators included caveats to rely on your own experience. As Lewes summarised:

even when Science shall have established laws … to accurately express the general value of each substance of food … the differences among individuals are so numerous, and often so profound, as to justify the adage, ‘one man’s meat is another man’s poison.’ (121)

Ultimately, we see invocations to ‘slow down’ and respect the intermittent tempos of ‘gastric time’. Defaecation, *The Family Physician* declared, ‘is not a thing to be done in a hurry. Many of us spend an hour over dinner and never grudge the time, but five minutes spent over an equally important matter is all too long’ (192). The slow intermittence of gastric time resisted the ‘fast-paced, high-speed network society’ of the nineteenth century, reminding us that we are subject, as Robert Hassan points out, to many ‘rhythms, and cycles and tempos that do not synchronise with the mechanical and unerring system of time based on numbers’ (2007, 37–38).

Indeed, gastric time sits alongside ‘grief time’ and ‘convalescent time’ as temporal experiences that deviated (however reluctantly) from the modern, normative, linear language of the clock. For Dana Luciano (2007), grief time was a response to a ‘new time-consciousness’, a ‘new shape of time’ (2) that emerged during the nineteenth century, brought about by technological development—standardised clocks, railroad timetables, telegraphic connection—and by social and cultural reorientations: new, capitalist labour expectations, and the rise of the discipline of history, with its narratives of progress from past to present. Grief time, by contrast, centred ‘the slow time of deep feeling’ (2) and was ‘collective rather than productive, repetitive rather than linear, reflective rather than forward-moving’ (6).

Hosanna Krienke (2021) likewise develops the critical concept of ‘convalescent time’, a temporality that was, for the Victorians, fitful, indeterminate, and agonisingly non-linear. Convalescence provided a time-scale that was removed from the pressures of nineteenth-century productivity, she argues, located physically in the spaces of convalescent homes, conceptually in the practices of domestic convalescence, and narratively in the forms of Victorian convalescent novels.

The ambling times of grief and convalescence encourage a kind of self-reflection and bodily attunement that speaks to another form of time: ‘crip time’—the tempos of disability and chronic illness. For Ellen Samuels, crip time ‘requires us to break our bodies and minds to new rhythms, new patterns of thinking and feeling and moving through the world …. crip time means listening to the broken languages of our bodies, translating them, honoring their words’ (2007, n.p). Such ulterior embodied understandings of time might sit productively alongside ‘gastric time’ as an avenue for reflecting on chronic gut problems like inflammatory bowel disease.

## The sympathetic stomach

The notion of ‘honouring the words of our bodies’ resonates with the ambit of gastric time as a temporal experience that was entangled with ideas about the connection between the gut and the brain. A long tradition of using digestion as a semantic proxy for cognitive processes—the ‘digestion’ of information—was given renewed significance by those who insisted that ‘brain work’ and ‘gut work’ were dynamically linked. In 1869, English physician Thomas Hawkes Tanner connected ‘mental depression’ with functional derangement in those organs that were ‘connected with the nutritive processes’ (499),⁠ suggesting that a problem with alimentation could have far-reaching consequences for an individual’s emotional health and cognitive ability. A correlation between ‘brain-workers’ and dyspeptics had been widely accepted for some time, pairing the connection between the stomach and the brain over and above other sympathetic connections like a primary telegraph line. Dyspepsia was thought to ‘interfere with intellectual work, and impede the expression of thought’ (*The Family Physician* 1883, 358), cause bad dreams, and hamper emotional regulation. Meanwhile, too much intellectual labour, overwork, and strong emotions (especially those caused by shock and stress) were catalogued as disrupting digestion and eventually leading to dyspepsia—a vicious cycle.

Edited collections on the cultural histories of the gut have highlighted its entanglements with moral politics and the history of emotions (Forth and Carden-Coyne 2005; Mathias and Moore 2018). In his experiments on Alexis St Martin, William Beaumont had noted that strong mental emotions such as anger and impatience would impair St Martin’s digestive capabilities by arresting the secretion of gastric juice and prompting an influx of bile, providing a physiochemical rationale for a long-standing association between disagreeableness and the so-called bilious temperament. John Timbs repeated the finding for a popular audience in his *Hints for the Table; or the Economy of Good Living* (1838), where it sat alongside aphorism, metaphor, and literary allusion in a hodgepodge of dietetic advice. Indeed, the multivocal landscape of gut health was such that emergent physiological knowledge decorated the evidentiary weight of metaphor and idiom—what one writer called the ‘concentrated wisdom of nations’. Writing for the *New Monthly Magazine* (1825) they reflected on the ‘intimate sympathy between body and mind’, concluding that the stomach and not the head was ‘unquestioningly the seat of thought in the human subject’ (Insubordination of Modern Stomachs, 36). We need only look at our tradition of metaphor to relocate the stomach within a long history of human emotional experience, they argued. Dubbing the stomach our ‘intellectual citadel’, they also drew readers attention to its anatomical position in the centre of the body:

What should we say of a people who should establish their capital upon an extreme frontier instead of the centre and heart of a country, and why should we suppose nature to be less provident in this respect than men? (36)

Here they employ a variation of the body-politic metaphor (in which body and nation are superimposed) to amplify the role of the stomach in the organic economy.

In his theory of nervous dyspepsia, Edward Johnson would also invoke the body-politic metaphor to conceptualise the sympathy between the mind and the gut in spatial terms. Positioning the brain and the stomach as two English cities, he swapped his Divine watch metaphor for that of the telegraph, asking his readers to imagine telegraphic wires stretched between London and Bristol to demonstrate the disordering of the nervous system through sympathy:

There are wires stretched between London and Bristol. Certain causes are made to operate on the London end of these wires, and an effect is instantly produced at the Bristol end, corresponding to the *kind* or nature of the cause set up at the London end. Now the brain is London, and the stomach is Bristol, and the gastric portion of the pneumo-gastric nerve is the wire. (1849, 102)

The near immediate transmission of information over spatial distance embodied by the telegraph provides Johnson with a way of explaining pathology in the body: ‘as the stomach may be disordered by brain-intemperance, so the brain may be disordered (and sometimes is) by stomach-intemperance’, in the same way that ‘a communication may be propagated along the electric wires from London to Bristol, and an answering communication be propagated back again from Bristol to London’ (104).

Despite the bidirectional flow of impulses, Johnson insisted that even if the stomach is irritated, this irritation still issues from the brain: ‘…as sometimes happens, the stomach may become deranged from irritation set up in a distant organ—that irritation being propagated to the brain, and from the brain to the stomach. Still even here the disordered stomach arises *directly* from the brain’ (114–15). For Johnson, the brain is like a co-ordinating telegraph operator, receiving information from around the body and tasked with sending it on to other organs.

Others however used the inherent omnidirectionality of telegraphic communication to privilege the expansive influence of the stomach, placing the two organs in competition and playing out tensions of bodily agency and autonomy. Such questions were brought into focus for a popular audience in 1853 when writer and Middle Temple barrister Sydney Whiting anonymously published *Memoirs of a Stomach*, purporting to be the autobiographical musings of that bodily organ.

### Memoirs of a stomach

Part medical lecture, part domestic health manual, part satirical fiction, Whiting’s hybrid text joined the diverse and burgeoning genre of dietetics—texts concerned with the relationship between food and health. Narrated by ‘Mr Stomach’, *Memoirs of a Stomach* manipulates the autobiographical genre to explore the primacy of the stomach within the wider bodily economy.

The book went through at least eleven editions and received numerous positive reviews. The *News of the World* called it ‘the most witty, learned, and truthful book’ they had seen for a long time, whilst the *Sun*, the *Sunday Times*, and the *Spectator* praised both its scientific content and its writing style. Although undoubtedly satirical, for many it provided an accessible avenue for medical knowledge; as a writer for *The Athenaeum* observed, ‘this is a humorous account of the functions of the stomach—and it may be made, perhaps, the vehicle for information for those who would not read a scientific treatise on the subject’ (The Memoirs of a Stomach 1853, 1386). Another reviewer went as far as to say that the ‘advice of this well-written book would, if strictly followed, in nine cases out of ten, supersede the necessity of physic or physician’ (Qtd in *Heliondé or Adventures in the Sun*, n.p).

Thus, *Memoirs of a Stomach* occupies an uncertain position in the literary marketplace, reflecting the emergence of forms of popular science that were read for ‘rational recreation’ (or entertainment value rather than solely education) (Dawson et al. 2004) as well as indicating the sheer variety of venues that propagated discussions of gastrointestinal health. Such discussions were regularly aired in family periodicals, cookery books, and domestic health manuals, as well as in novels and poems; they even formed the basis for commercial advertisements selling patent medicines where the telegraphic body often made an appearance. An advertisement for Mother Seigel’s curative syrup, for example, described the symptoms of nervous illness in the following words: ‘it is as though a nerve had somehow broken loose from its fastenings and was being shaken and slatted about, like a telegraph wire in a gale’ (The Strongest Are Safest, 1897, 34). They asserted that the syrup could restore ‘the strength and vigour of the whole body’ by targeting the digestion, thus placing emphasis on the stomach as an arbiter of nervous health.

Whiting also drew on the idea of the telegraphic body. Mr Stomach claims that between himself and his ‘help-mate’ Mr Brain there ‘was established a double set of electric wires’, through which he would often send and receive ‘news’ and ‘information’ (21). Whiting plays on the imaginative parallels between cognitive and gastric processes by having Mr Stomach complain that when Mr Brain did not adequately ‘digest’ information before sending it on to him, he would develop a stomach ache. This drew on a prominent cultural narrative that juxtaposed reading with eating but also echoed medical treatise that saw the ‘information overload’ of modern life as gastrically debilitating. Influential American physician Silas Weir Mitchell had famously connected the mental pressures of modern labour expectations with diseases like nervous dyspepsia; ‘the wearing, incessant cares of overwork, of business anxiety, and the like’, he writes, ‘produce directly diseases of the nervous system, and are also the fertile parents of dyspepsia’ (1871, 51). Others agreed, identifying dyspepsia as a condition that might be precipitated by mental strain. Arthur Leared, for example, insisted that:

the origin and connections of the pneumogastric nerves with the cerebro-spinal centres … show how it is that purely mental impressions, such as are caused by bad news or fright … may disturb digestion. (1879, 622)

Johnson had also characterised dyspepsia in this way, arguing that it was scarcely found in the labouring classes but ‘almost universal’ in the middle classes, who live by the sweat of their brain and not their brow, and in the upper classes too, who live ‘under the perpetual influence of strong nervous excitement’ (1849, 106).

Whilst Mitchell and Leared were writing later in the century (and Johnson was writing from a contested position as a practitioner of what was often viewed as a popular but unorthodox treatment—hydropathy), Whiting was indebted to similar ideas circulating in the medical press in the 1850s. He had certainly read Sir James Eyre’s *The Stomach and its Difficulties*, published the preceding year, which he footnotes throughout *Memoirs*, and which became something of a stylistic model. Eyre had dubbed the stomach both an ‘invaluable slave’ and a ‘mischievous and dangerous, because powerful, Despot’ (1852, vii), a characterisation that Whiting makes literal. He describes the expansive power of the stomach, who like a despot is able to control ‘the little body of which [he is] the centre’, by inciting (in response to unpalatable milk) ‘[his] neighbouring arms and legs to kicks and contortions’, and by suggesting ‘shrill cries’ ‘to the small voice that dwelt upstairs’ (12). Moreover, he insists that he resides in the drawing room, whilst Mr Brain lives in the attics—a comment that, whilst describing actual anatomical positions, also alludes to a domestic and spatial hierarchy (servants’ quarters often being located in the attics). At other moments, Mr Stomach uses the language of bondage to locate himself within what he calls the ‘somatic household’: he speaks in terms of ‘labour’, characterises the person in whom he resides as his ‘master’, and describes being whipped like a cab-horse, and being treated like a ‘galley-slave’ (76).

The vexed ontological position of the stomach is reinforced by the novel’s paratexts. Despite giving literal voice to the stomach in his choice of narrator, Whiting mediates Mr Stomach via sometimes contradictory editorial footnotes that work to undermine the stomach’s intellectual authority (‘we cannot but deplore this unnecessary divergence of our author from his starting point’ (94)). In the same moment, however, these footnotes reiterate the fullness of his agency by demonstrating Mr Stomach’s possession of independence of ‘mind’.

Elsewhere, the editor’s footnotes serve to corroborate Mr Stomach’s assertions about diet by supplementing them with contemporaneous medical knowledge. Such editorialising bolsters the stomach’s narrative agency. By creating the pretence of editor-author disagreements and offering stylistic criticism of Mr. Stomach’s writing, the footnotes muddy the boundaries between author, editor, and protagonist, meanwhile conveying—stylistically—the idiosyncrasies of digestion as a bodily process and the contested autonomy of the stomach.

Whiting uses literary conceits to bring complex ideas to a lay audience. Eyre’s assertion in his medical textbook that the stomach is ‘very like in shape to the Caledonian bagpipe’ is in Whiting’s novel transformed into a playful gastric mythology: ‘Ye Legend of Ye Bagpipe’, in which an early Nord King invading Scotland punishes a would-be assassin by removing his digestive organs and fashioning them into an instrument by which could be heard the screams of the tortured man forevermore. This is an image that does double duty in communicating anatomical information and immortalising the pain and suffering induced by digestive distress.

The understanding of gastric sympathy that underpins Eyre’s book—itself indebted, as Eyre acknowledged, to writers like Abernethy—becomes for Whiting not a physiological sympathy, but a felt empathy or compassion designated by Mr Stomach’s characterisation of his relationship with Mr Brain as one of neighbours, friends and relatives, and an ‘adjacent brotherhood’ (37). Mr Stomach asserts, ‘often when [Mr Brain] has received some unwelcome intelligence, I have *refused* to digest out of pure sympathy; and when occasionally I grew morose and refused to work, he too grew irritable and petulant’ (22). Whiting plays on the double meaning of sympathy to designate both ‘similarity of action between totally different parts of the body’ (Bower 1842, 191) and to refer to an imaginative process of shared emotional experience. Such language mediates readers’ knowledge of gut health in ways that emphasise the role of both material and immaterial connection.

## The gastric telegraph: Connection and disruption

In a series of character sketches entitled ‘Our Village’, a writer for the British illustrated literary magazine *Once a Week* sung the praises of a squire with the ‘habits of a dormouse’. Sir Edward Worsell had a remarkable penchant for sleep and was, in their opinion, all the better for it, never spending more than his ‘income’ of ‘nerve power’. Worsell provides a mirror of contrast through which readers might perceive their own epoch as one of life speedily lived. ‘This is a fast age,’ they write, ‘we want our blood to go through its veins at railway speed; we want to use our nerve system as busily as the wires of the telegraph are used’ (1872, 77–78). This echoed ubiquitous sentiments in the British and American periodical presses commenting on the untenable speed of modern life.

As Janet Oppenheim (1991) has noted, the idea that the human body had a finite amount of ‘nerve force’ that could be depleted dominated medicine from the mid-century. Such a notion, which was borrowed from energy physics, articulated a set of anxieties about the fitness of human bodies for modern life and labour. The digestive system played a central but often overlooked role in this model because it was a key site of replenishment but also of energy consumption. Consequently, faulty digestion was liable to put strain on the system by diverting too much energy from other bodily processes such as cognition. Likewise, too much nerve force spent in the pursuit of ‘brain-work’ could rob the digestive system of energy and so damage it, causing inflammation that might rebound on the rest of the body and lead to a variety of conditions often marshalled under the umbrella term: dyspepsia.

The functional and practical complications of modern communication technologies like the telegraph enabled writers to conceptualise dyspepsia as a complaint associated with disruption and delay. A writer identifying themselves as ‘Christian at work’, for example, envisaged the nervous system in 1888 as a complex telegraphic network by which messages from ‘the headquarters in the brain are sent to the minute stations in the extremities’. Writing for the *Washington Post*, they asserted that when the system is in disarray it has a profound effect on the ‘digestion’ of information and emotion: ‘when we are glum, and dismal, and low-spirited, the telegraphic apparatus is out of order’. They conceptualise this breakdown by invoking the material structures of telegraphy: ‘it is as when telegraph poles are shaky or wires entangled or crossed, or currents irregular …. the wires become entangled and we are irritable, sulky, ill-tempered or angry …. [we] misinterpret and misunderstand’ (11).

Many writers likewise framed ill-health in terms of miscommunication. In the same year, Scottish physician Sir Thomas Lauder Brunton had insisted that in health ‘there appears to be a sense of perfect intelligence conveyed from the stomach to the encephalic centre’—paralleling the ideal of a city connected by telegraph wires (60). In poor health, however, this ‘perfect intelligence’ was compromised. Such a notion hinged on the tenet that both nerve and telegraph transmitted, not mere impulse, but coded ‘message’, an idea borne out for a popular readership in Spencer Thomson MD’s series on travelling for health, in which he described hunger as the activity of impoverished organs ‘telegraph[ing] to the stomach for supplies’ (1861, 37).

Alexander Engel argues that the widespread use of the telegraph in the nineteenth century meant that ‘for the first time in history, information could travel much faster over vast distances than goods and people’ (2015, 287). This fast circulation of knowledge and slow circulation of goods paralleled the relationship between nervous stimuli and the digestion of food, a parallel invoked to explain the phenomenon of overeating: ‘No doubt, the stomach, as it became gradually distended informed the brain through its nerves, that food was on its way. But still, this was not sufficient and the appetite remained unappeased’ (Brunton 1888, 59). The fast action of the nerves outstrips the slow digestion of food, suggesting that alimentary problems could be explained by the inherent disparity between the physiological time-scales of the nervous and digestive systems made plain by the model of the telegraph.

The telegraphic metaphors that underpinned intra-bodily communication, however, also revealed the differences between nature and technology. As one writer for *The Spectator* remarked in 1866:

Scientific men assert that the nerves of the human body are, to all intents and purposes, a telegraphic apparatus, in which however the nervous agent, or equivalent of electricity, travels along the nervous cable indefinitely more slowly than electricity along the wire … if then the human nerves be carriers of information which are infinitely more tardy … may we not fairly suppose [that they] are liable not only to the same class of perturbations as the magnetic cable itself, but to more and greater. (994)

The slower nature of nervous communication placed it in danger of greater disruptions than even the recently laid submarine cable of the Atlantic telegraph, which itself had encountered severe problems, taking five attempts over nine years to finally produce a reliable and lasting connection. Just as the telegraph only supplied an illusion of connectivity that was not always tenable, its use as an analogy to think about neurogastric networks emphasised not only the healthy body’s quasi-telegraphic communication but also its vulnerability to delay, disruption, and pathological incoherence. Here the telegraph offers a frame of reference that illustrates how, even in health, the body can let us down—how the disparity between mechanical ideal and organic reality exposes the mismatch between human physiology and modern labour expectations modelled on the efficiency of the factory.

### Telegraphic thinking

George Lakoff and Mark Johnson claimed more than 40 years ago that the way we *speak* about the world shapes the way we think about it, experience it, and relate to it. Whilst metaphor allows us to understand one thing in terms of another—the gut-brain axis in terms of the telegraph network—it may also ‘keep us from focusing on other aspects of the concept that are inconsistent with that metaphor’ (1980, 18). Thus, turning a critical lens on how we speak about our bodies is paramount. The full complexity of what we now know as the gut-brain-microbiome axis falls beyond the scope of what telegraphy can represent; nevertheless, the metaphor of the telegraphic body emphasises a dynamic kinship between ‘reading and eating’ and ‘thinking and digesting’ that might help us reflect in new ways on the prevalence of comorbidities like, for example, anxiety disorders and inflammatory bowel disorders. How might taking a holistic approach to our mental and nutritional health serve us? The ascendency of microbiome studies has ushered in a new roster of ecological metaphors that speak to emerging cultural concerns about deforestation, climate change, and lost or damaged relationships with microbial communities. Such figurations enable and occlude ways of knowing and thinking about heath in entirely new ways.

A side effect of the COVID-19 pandemic was a refocusing on our cultures of labour and a widespread (though for some brief) practice of flexible working. At a time still largely characterised by overwork, burn out, and their attendant mental health crises, the telegraphic body might help us reorient ourselves in relation to past lessons poorly learnt. Whilst the metaphor unhelpfully encourages us to view health within a narrow box of ‘working order’, it, at the same time, provides an intermittent, idiosyncratic understanding of the temporal-spatial parameters of the body that is barely contained by its linguistic framework. Reconsidering health in the context of ‘gastric time’ might sit productively alongside Kristin D. Hussey’s call for a ‘rhythmic history’ approach to histories of health and medicine—an approach that concerns itself with environment and the temporalities of the body, with ‘the way time is lived, embodied and effects bodily health and disease’ (2022, 3).

Ultimately, the figurative language of the telegraphic body helped to redefine a range of bodily ailments as problems of time, space, and communication. Diverse venues with ranging audiences jointly constructed the idea of the ‘telegraphic body’, from orthodox medical voices to more fringe practitioners, from domestic health manuals, self-help tracts, and cookery books, to periodical essays, advertisements, and satirical fiction. This figuration was one among many ways that people conceptualised ‘dis-ease’ within the body, but it was one that enabled commentators—through the symbolism of the telegraph as an ascendent communications technology that was central to labour and commerce—to reflect on anxieties about the demands of modern life on the digesting body. The limits of such analogical thinking prompt us to consider not only the unfitness of our bodies for industrial modernity but the unfitness of industrial modernity for our bodies.

## Declarations

### Endnotes

^1^ See for example: John S. Main, Cerebral dyspepsia. *British Medical Journal*, 10 Feb 1883: 249; Henry Browne, Oral, duodenal, and gastric dyspepsia. *British Medical Journal*, 30 Dec 1865: 679–683; Anon, An epitome of current medical literature: Nervous dyspepsia. *British Medical Journal*, 15 Jan 1898: 9.

## References

[CR1] Abernethy, John. 1830. *Common sense, or the Abernethian code of health and longevity.* London: Printed for the Booksellers.

[CR2] Anon. 1825. Insubordination of modern stomachs. *The New Monthly Magazine and Literary Journal* 13(49): 35-39.

[CR3] Anon. 1859. A physician’s note-book. *The Critic* 19(483): 342-344.

[CR4] Anon. 1865. Digestibility of food. *The London Reader of Literature, Science, Art, and General Information* 6(138): 284.

[CR5] Anon. 1872. Our village—IV. *Once a Week* 10(239): 77-78.

[CR6] Anon. 1879. ART. III – 1. Manual of human and comparative histology. *The Edinburgh Review* 149: 58-83.

[CR7] Anon. 1883. *The family physician: A manual of domestic medicine,* vol. II*.* London: Cassell and Company.

[CR8] Anon, 1897. The strongest are safest. *Strand magazine: an illustrated monthly* 13(78): 34.

[CR9] Anon. 1853. The memoirs of a stomach. *The Athenaeum* (1360): 1386.

[CR10] Anon. 1866. The awakening of the cable. *The Spectator* (1993): 993-995.

[CR11] Bell, Robert. 1899. On the treatment of a stomach. *The Butterfly* 2(2): 135-141.

[CR12] Bower, Mark Noble. 1842. On sympathy. *The Lancet* 38(975): 191-192. 10.1016/S0140-6736(02)85127-3

[CR13] Brillat-Savarin, J.A. 1825. *A handbook of gastronomy,* A Lalauze (trans) 1825. London: J. C. Nimmo and Bain.

[CR14] Browne, Henry. 1865. On oral, gastric, and duodenal dyspepsia. *British Medical Journal* 2(261): 679-683.

[CR15] Brunton, T. Lauder. 1888. *Disorders of digestion: Their consequences and treatment.* London: Macmillan and Co.

[CR16] Cheyne, George. 1733. *The English malady, or a treatise of nervous diseases of all kinds, as spleen, vapours, lowness of spirits, hypochondraical, and hysterical distempers.* London: G. Strahan.

[CR17] Chico, Tita. 2018. *The experimental imagination: Literary knowledge and science in the British Enlightenment.* Stanford: Stanford University Press. 10.11126/stanford/9781503605442.001.0001

[CR18] Christian at Work. 1888. Nerves and moods: A complex system of telegraphy and cause of bad temper. *The Washington Post* (18 Nov 1888): 11.

[CR19] Cooper, Astley. 1835. *Lectures on the principles and practices of surgery.* 8^th^ ed. London: Edward Portwine and John Thomas Cox.

[CR20] Dawson, Gowan, Richard Noakes and Jonathan R. Topham. 2004. Introduction. In *Science in the nineteenth-century periodical* eds. Geoffrey Cantor, Gowan Dawson, Graeme Gooday, Richard Noakes, Sally Shuttleworth, and Jonathan R. Topham, 1-36. Cambridge: Cambridge University Press.

[CR21] Engel, Alexander. 2015. Buying time: Futures trading and telegraphy in nineteenth-century global commodity markets. *Journal of Global History* 10(2): 284-306. 10.1017/S1740022815000078

[CR22] Eyre, James. 1852. *The stomach and its difficulties.* London: J Churchill.

[CR23] Forth, Christopher E. and Ana Carden-Coyne. 2005. *Cultures of the abdomen: Diet, digestion and fat in the modern world.* New York: Palgrave Macmillan. 10.1057/9781403981387

[CR24] Foster, Russell. 2022. *Life time: The new science of the body clock and how it can revolutionize your sleep and health.* London: Penguin Life.

[CR25] Griffiths, Devin. 2016. *The age of analogy: Science and literature between the Darwins.* Baltimore: Johns Hopkins University Press. 10.1353/book.47914

[CR26] Hassan, Robert. 2007. Network Time. *In 24/7: Time and temporality in the network society* eds. Robert Hassan and Ronald E. Purser, 37-61. Stanford: Stanford University Press.

[CR27] Hussey, Kristin D. 2022. Rhythmic history: Towards a new research agenda for histories of health and medicine. *Endeavour* 46(4): 1-10. 10.1016/j.endeavour.2022.100846 10.1016/j.endeavour.2022.100846 36521301

[CR28] Johnson, Edward. 1849. *Results of hydropathy, or, constipation not a disease of the bowels, indigestion not a disease of the stomach.* London: John Wiley.

[CR29] Kennaway, James and Jonathan Andrews. 2019. "The grand organ of sympathy": "Fashionable" stomach complaints and the mind in Britain, 1700-1850. *Social History of Medicine* 32(1): 57-79. 10.1093/shm/hkx055

[CR30] Ketabgian, Tamara. 2011. *The lives of machines: The industrial imaginary in Victorian literature and culture.* Ann Arbour: University of Michigan Press. 10.3998/dcbooks.9544598.0001.001

[CR31] Kitchiner, William. 1821. *Peptic precepts: Pointing out agreeable and effectual methods to prevent and relieve indigestion, and to regulate and invigorate the action of the stomach and bowels.* London: Printed for S Bagster by J Moyles.

[CR32] Krienke, Hosanna. 2021. *Convalescence in the nineteenth century novel: The afterlife of Victorian illness.* Cambridge: Cambridge University Press. 10.1017/9781108953788

[CR33] Lakoff, George, and Mark Johnson. 1980. *Metaphors we live by.* Chicago: University of Chicago Press.

[CR34] Leared, Arthur. 1860. *The causes and treatment of imperfect digestion.* London: Churchill.

[CR35] Leared, Arthur. 1879. A neglected proximate cause of dyspepsia, with a new division of the disease. *British Medical Journal* 1(956): 622-23. 10.1136/bmj.1.956.622 10.1136/bmj.1.956.622 PMC2240030 20749184

[CR36] Lewes, George Henry. 1859. *The physiology of common life.* Vol I. Edinburgh: W. Blackwood.

[CR37] Liffen, John. 2013. Telegraphy and telephones. *Industrial Archaeology Review* 35(1): 22-39. 10.1179/0309072813Z.00000000014

[CR38] Luciano, Dana. 2007. *Arranging grief: Sacred time and the body in nineteenth-century America.* New York: New York University Press.

[CR39] Mathias, Manon, and Alison M. Moore. 2018. *Gut feeling and digestive health in nineteenth-century literature, history, and culture*. London: Palgrave Macmillan. 10.1007/978-3-030-01857-3

[CR40] Miller, Ian. 2011. *A modern history of the stomach: Gastric illness, medicine and British society, 1800 – 1950.* London: Routledge. 10.4324/9781315655574

[CR41] Mitchell, Silas Weir. 1871. *Wear and tear, or hints for the overworked.* Philadelphia: J. B. Lippincot and Co.

[CR42] Morus, Iwan Rhys. 2000. "The nervous system of Britain": Space, time, and the electric telegraph in Victorian Britain. *British Journal for the History of Science* 33(4): 455-475. 10.1017/S0007087400004210

[CR43] Musselman, Elizabeth Green. 2006. *Nervous conditions: Science and the body politic in early industrial Britain.* New York: State University of New York Press.

[CR44] OED. 2024. Dyspepsia (n.). *Oxford English Dictionary*. Online. 10.1093/OED/1784206738

[CR45] Oppenheim, Janet. 1991. *Shattered nerves: Doctors, patients, and depression in Victorian England.* Oxford: Oxford University Press. 10.1093/acprof:oso/9780195057812.001.0001

[CR46] Otis, Laura. 2001. *Networking: Communicating with bodies and machines in the nineteenth century.* Ann Arbour: University of Michigan Press. 10.3998/mpub.17323

[CR47] Rabinbach, Anson. 1992. *The human motor: Energy, fatigue, and the origins of humanity.* Berkeley: University of California Press.

[CR48] Samuels, Ellen. 2017. Six ways of looking at crip time. *Disability Studies Quarterly* 37(3) online: https://dsq-sds.org/index.php/dsq/article/view/5824/4684

[CR49] Saundby, Robert. 1894. Abstract of the Ingleby lectures on the common forms of dyspepsia in women. Lecture I. *The Lancet* 143(3681): 661-663. 10.1016/S0140-6736(01)67624-4

[CR50] Servitje, Lorenzo. 2021. *Medicine is war: The martial metaphor in Victorian literature and culture.* New York: SUNY Press.

[CR51] Tanner, Thomas Hawkes. 1869. *The practice of medicine.* Vol I. 6^th^ Edn. London: Henry Renshaw.

[CR52] Thomson, Spencer. 1861. Trips after health, and how to profit by them. *The Sixpenny Magazine* 1(1): 32-37.

[CR53] Timbs, John. 1838. *Hints for the table; or the economy of good living.* London: Kent and Co.

[CR54] Wenzlhuemer, Ronald. 2012. *Connecting the nineteenth century world: The telegraph and globalization.* Cambridge: Cambridge University Press. 10.1017/CBO9781139177986

[CR55] Whiting, Sydney. 1853. *Memoirs of a stomach; written by himself, that all who eat may read.* London: W. E. Painter.

[CR56] Whiting, Sydney. 1855. *Heliondé; or adventures in the sun.* London: Chapman and Hall.

[CR57] Whytt, Robert. 1765. *Observations on the nature, causes, and cure of those disorders which have been commonly called nervous, hypochondriac, or hysteric, to which are affixed some remarks on the sympathy of the nerves.* Edinburgh: J. Balfour.

